# Zinc finger transcription factor ZNF24 inhibits colorectal cancer growth and metastasis by suppressing MMP2 transcription

**DOI:** 10.1016/j.gendis.2025.101529

**Published:** 2025-01-10

**Authors:** Shuo Tian, Xia Chen, Jianzhong Li

**Affiliations:** a Department of Biochemical Pharmacy, Naval Medical University, Shanghai 200433, China; b Key Laboratory, Yangpu Hospital, Tongji University School of Medicine, Shanghai 200090, China

Among the most common malignancies, colorectal cancer (CRC) has a high incidence and mortality rate.^1^ Matrix metalloproteinase 2 (MMP2) plays an important role in the migration and invasion of malignant tumor cells.^2^ Although many trans-activating factors of MMP2 have been identified, relatively little is known about MMP2 transcriptional inhibition. In this study, we found that zinc finger transcription factor ZNF24 (zinc finger protein 24) may be a novel transcriptional repressor of MMP2 expression.

ZNF24 was originally identified while trying to discover a new C2H2 zinc finger protein in the hematopoietic system.^3^ Our previous analysis has revealed that ZNF24 is a pleiotropic factor that has a role in hematopoiesis, brain development, and cancers.^4^ Although studies have explored the paradoxical role of ZNF24 in the initiation and development processes of various cancers, the precise role and underlying mechanism of ZNF24 in CRC remain largely unclear.

To determine the relevance of ZNF24 in CRC, we first evaluated whether the expression of ZNF24 was altered in CRC by analyzing TCGA data from public databases. We found that ZNF24 was down-regulated in colorectal adenocarcinoma, while there was no significant difference in mucinous adenocarcinoma compared with normal tissues (Fig. S1A). To further investigate the expression status of ZNF24 in CRC, we compared the expression of ZNF24 mRNA and protein in 15 cases (13 cases of colorectal adenocarcinoma and 2 cases of mucinous adenocarcinoma) of CRC fresh tissues (T) and their adjacent normal tissues (N). As shown in Figure S1B and C, the expression of ZNF24 mRNA and protein was down-regulated in (11 out of 13) colorectal adenocarcinomas in comparison to adjacent non-cancerous tissues, while no significant differences were observed in 2 mucinous adenocarcinomas.

To investigate the potential function of ZNF24, we used functional gain- and loss-of-function methods to evaluate the role of ZNF24 in CRC. As shown in Figure S2A and B, the constructed lentivirus system could effectively up-regulate or down-regulate the expression of ZNF24 in HCT116 and SW620 cells. CCK-8 cell proliferation assay showed that ZNF24 overexpression significantly inhibited the proliferation of HCT116 and SW620 cells, while ZNF24 knockdown significantly promoted cell proliferation (Fig. S2C). Consistent with the CCK-8 assay results, ZNF24 overexpression significantly reduced colony formation ability, while ZNF24 knockdown significantly improved colony growth and colony formation abilities (Fig. S2D). Consistently, flow cytometric analysis results showed that ZNF24 overexpression caused a significant increase in the percentage of G0/G1 phase HCT116 and SW620 cells, while ZNF24 knockdown caused a significant decrease in the percentage of G0/G1 phase HCT116 and SW620 cells (Fig. S2E). In addition, the effects of ZNF24 on the senescence of HCT116 and SW620 cells were analyzed by β-galactosidase staining. The results showed that ZNF24 overexpression could significantly promote the senescence of HCT116 and SW620 cells, while ZNF24 knockdown could significantly reduce the proportion of cell senescence (Fig. S2F), suggesting that ZNF24 inhibits the growth of CRC cells by mainly inducing cell senescence. To evaluate the role of ZNF24 in modulating the growth of CRC cells *in vivo*, we established a xenograft mouse model by transplanting ZNF24 overexpression or knockdown HCT116 cells. As shown in Figure 1, ZNF24 overexpression significantly inhibited the growth of HCT116 cell grafts (Fig. 1A–C), while ZNF24 knockdown significantly promoted the growth of HCT116 cell grafts (Fig. 1D–F).

**Figure 1:**
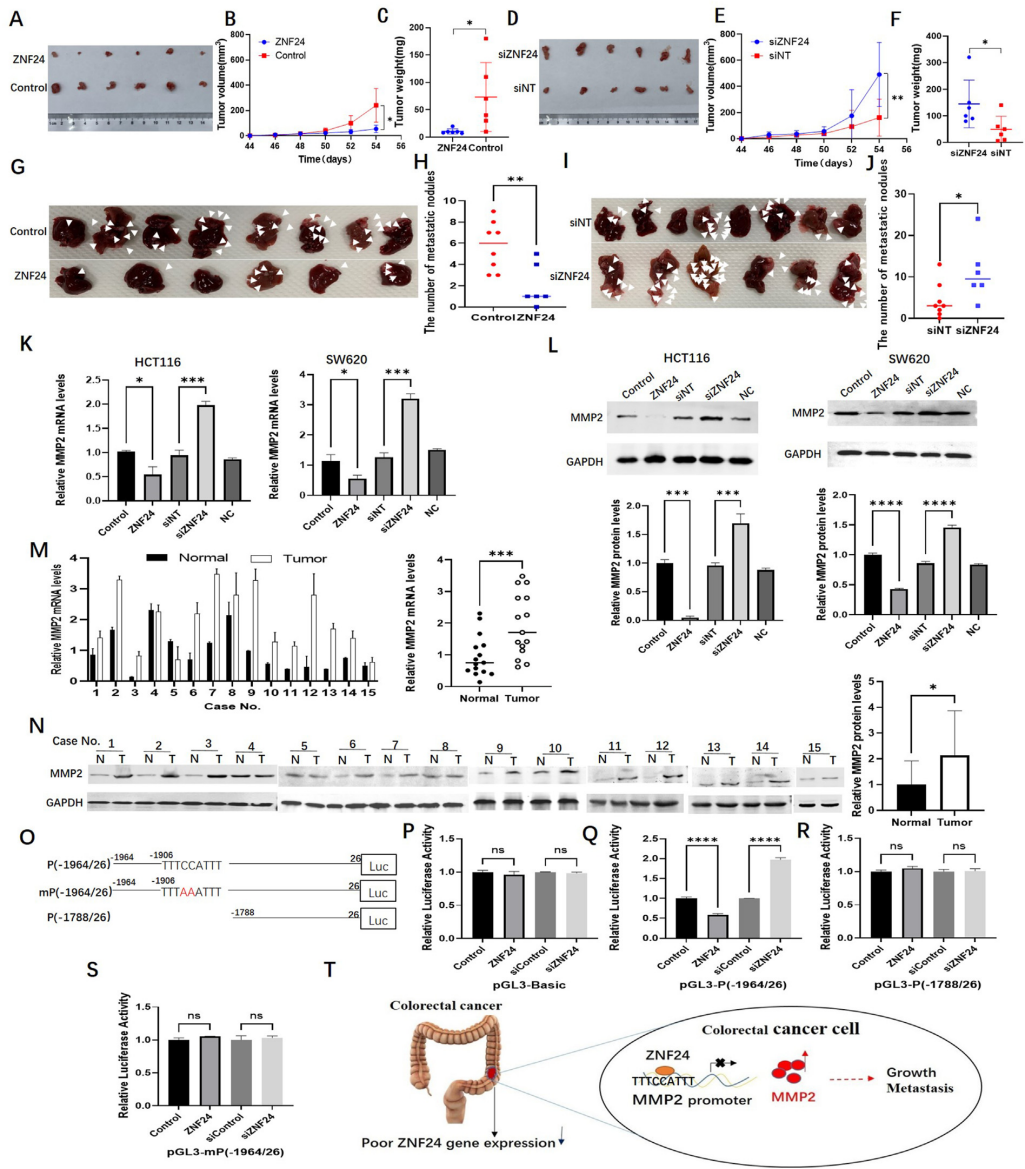
ZNF24 inhibits growth and metastasis of colorectal cancer and inhibits MMP2 expression. **(A)** Gross photos of xenograft tumors were removed 8 weeks after subcutaneous injection of ZNF24 overexpressed or control HCT116 cells (*n* = 6). **(B)** The tumor volume of subcutaneous xenografts at the indicated days after injection. **(C)** The scatter plots showing the weight of removed tumors. **(D)** Gross photos of removed xenograft tumors 8 weeks after subcutaneous injection of ZNF24 knockdown or control HCT116 cells (*n* = 6). **(E)** The tumor volume of subcutaneous xenografts at the indicated days after injection. **(F)** The scatter plots showing the weight of removed tumors. **(G)** Images of liver metastases in mice after 6 weeks of intrasplenic injection of overexpressed ZNF24 or control HCT116 cells. Arrows, macroscopically visible liver metastasis. **(H)** The scatter plots showing the quantification of macroscopic metastatic foci in the liver of mice. **(I)** Images of liver metastases in mice after 6 weeks of intrasplenic injection of ZNF24 knockdown or control HCT116 cells. **(J)** The scatter plots showing the quantification of macroscopic metastatic foci in the liver of mice. **(K, L)** Quantitative reverse transcription PCR (K) and western blotting (L) were performed to determine the expression level of MMP2 in HCT116 and SW620 cells infected with ZNF24 overexpression and interference lentivirus. **(M)** ZNF24 and MMP2 expression levels were inversely correlated in pairs of malignant and adjacent normal human tissues. ZNF24 was inversely correlated with MMP2 mRNA expression in 10 of the 11 ZNF24 down-regulated CRC pairs (samples 1, 2, 3, 6, 7, 8, 9, 11, 12, and 13). Additionally, MMP2 was up-regulated in 12 of the tumor (T) samples compared with adjacent normal (N) tissues (samples 1, 2, 3, 6, 7, 8, 9, 10, 11, 12, 13, and 14). **(N)** ZNF24 was inversely correlated with MMP2 protein expression in 10 of the 11 CRC ZNF24 down-regulated CRC pairs (samples 1, 2, 3, 6, 7, 8, 9, 11, 12, and 13). Strikingly, MMP2 was up-regulated in 13 of the tumor (T) samples compared with adjacent normal (N) tissues (samples 1, 2, 3, 6, 7, 8, 9, 10, 11, 12, 13, 14, and 15). **(O)** Schematic diagram of MMP2 promoter fluorescence reporter vector. **(P)** ZNF24 overexpression and interference had no significant effect on the fluorescence activity of pGL3-Basic. **(Q)** Overexpression of ZNF24 significantly inhibited the activity of MMP2 promoter (−1964/26), while ZNF24 interference significantly promoted the activity of P(-1964/26). **(R)** ZNF24 overexpression and interference had no significant effect on the activity of P(-1788/26). **(S)** ZNF24 overexpression and interference had no significant effect on the activity of mutant promoter mP(-1964/26). **(T)** The hypothetical working model. ZNF24 represses MMP2 transcription by directly binding to the 9-bp fragment of the MMP2 promoter and functions as a negative regulator of colorectal cancer growth and metastasis.

Cell scratch assay (Fig. S3A) showed that ZNF24 overexpression inhibited wound healing in HCT116 and SW620 cells, while ZNF24 knockdown promoted wound healing. Consistent with the results of the wound healing assay, transwell migration and invasion assays showed that ZNF24 overexpression significantly inhibited cell migration and invasion, while ZNF24 knockdown significantly promoted cell migration and invasion (Fig. S3B, C). To further verify the effect of ZNF24 on the migration and invasion of CRC cells *in vivo*, a liver metastasis model was generated by spleen injection of ZNF24 overexpression or knockdown HCT116 cells in nude mice. As shown in Figure 1, ZNF24 overexpression significantly inhibited the liver metastasis of HCT116 cell grafts (Fig. 1G, H), while ZNF24 knockdown significantly promoted the liver metastasis of HCT116 cell grafts (Fig. 1I, J).

The finding that ZNF24 is down-regulated in CRC and inhibits CRC cell growth and metastasis prompted us to determine the mechanism of ZNF24. By mining the transcript profiling data of ZNF24 knockdown or overexpression in HEK-293T cells and the previous work,^4^ we found that MMP2 was a potential target of ZNF24. To investigate the relationship between ZNF24 and MMP2 in CRC, we analyzed the MMP2 mRNA and protein levels in HCT116 and SW620 cells with transient overexpression or knockdown of ZNF24. As expected, MMP2 mRNA and protein levels were down-regulated with ectopic overexpression of ZNF24 (Fig. 1K, L). Consistently, the expression of MMP2 increased in transient ZNF24 knockdown HCT116 and SW620 cells compared with controls (Fig. 1K, L). To further confirm the correlation between ZNF24 and MMP2 in CRC *in vivo*, the correlation between the ZNF24 mRNA and protein expression levels and MMP2 was further studied in 15 human CRC tissues. As expected, ZNF24 mRNA and protein levels were down-regulated in CRC compared with that of adjacent carcinoma (Fig. S1B, C), while the expression level of MMP2 in CRC was significantly up-regulated compared with that of adjacent carcinoma (Fig. 1M, N). The strong inverse correlation between ZNF24 and MMP2 expression was observed *in vitro* and *in vivo*, suggesting that ZNF24 may be a potential tumor suppressor that may negatively regulate MMP2 expression in CRC.

To verify the regulatory role of ZNF24 and MMP2 in CRC, we performed rescue experiments by inducing MMP2 expression in ZNF24 overexpressing cells. As expected, exogenous expression of MMP2 restored proliferative ability in ZNF24-overexpressing CRC cells through the CCK-8 assay (Fig. S4A). In an *in vitro* metastasis model, overexpression of MMP2 rescued the migration and invasion abilities in ZNF24-overexpressing CRC cells (Fig. S4B, C). Together, these results suggest that the repression of MMP2 expression is involved in ZNF24-mediated CRC progression and invasion.

ZNF24 is a transcription factor with four C2H2 zinc finger motifs that encode a putative DNA-binding domain. Therefore, we investigated the ability of this transcription factor to inhibit MMP2 transcription. To identify the essential region of the MMP2 promoter for ZNF24-mediated repression, we tested the ability of ZNF24 to repress full-length (−1964/26) or truncate (−1788/26) MMP2 promotor-driven luciferase reporter genes (Fig. 1O). ZNF24 overexpression was able to significantly repress the P(-1964/26) luciferase reporter gene, but not the P(-1788/26) luciferase reporter gene (Fig. 1Q, R). Consistently, ZNF24 knockdown was able to significantly promote the P(-1964/26) luciferase reporter gene, but not the P(-1788/26) luciferase reporter gene (Fig. 1Q, R), leading to the conclusion that the necessary regulatory elements required for ZNF24-mediated repression are located in the −1964/-1788-bp promoter region.

Next, we searched whether the −1964/-1788 promoter region contained the known ZNF24 binding sites. The 9-bp fragment TTTCCATTT (−1906/-1898) was found, similar to the VEGF binding site GCTTTCCATTT.^5^ To further investigate whether the candidate binding sequence TTTCCATTT was the key sequence for binding ZNF24 and MMP2 promoter, we examined the effect of ZNF24 on transcriptional activity of mutated type MMP2 promoter mP(-1964/26) with mutating key sequence TTTCCATTT to TTTAAATTT. As demonstrated in Figure 1S, the repression (or activation) of mutated MMP2 promoters by ZNF24 overexpression (or knockdown) was abolished compared with that of wild-type promoters. Taken together, these data suggest that ZNF24 can directly bind to the MMP2 promoter, and the key sequence of its binding site is TTTCCATTT.

In summary, ZNF24 represses MMP2 transcription by directly binding to the 9-bp fragment of the MMP2 promoter and acts as a negative regulator of CRC growth and metastasis (Fig. 1T).

## Ethics declaration

The study was approved by the Committee on Ethics of Medicine of Naval Medical University (approval number: 2022-025).

## Funding

This work was supported by grants from the 10.13039/501100001809National Natural Science Foundation of China (No. 81570557, 30871353).

## CRediT authorship contribution statement

**Shuo Tian:** Investigation. **Xia Chen:** Writing – review & editing, Resources, Conceptualization. **Jianzhong Li:** Writing – review & editing, Writing – original draft, Validation, Funding acquisition, Data curation, Conceptualization.

## Conflict of interests

All authors declared no conflict of interests.
