# Supplementary material for: Zinc finger transcription factor ZNF24 inhibits colorectal cancer growth and metastasis by suppressing MMP2 transcription
Source: Genes Dis. 2025 Jan 10;12(5):101529. doi: 10.1016/j.gendis.2025.101529 (PMC12164014; doi:10.1016/j.gendis.2025.101529)
Supplement: Multimedia component 1 [file mmc1.docx]

**Supplementary Materials and methods**

**Materials and Methods**

**Cell culture and lentivirus-based overexpression and knockdown**

Colorectal cancer HCT116, SW620 and normal human colon mucosal epithelial (NCM460) cell lines were obtained from the American Type Culture Collection (ATCC, USA) and cultured in DMEM medium supplemented with 10% fetal bovine serum (FBS) and 1% penicillin/streptomycin at 37°C and 5% CO_2_. Lentiviruses were generated by transfecting overexpression, knockdown and control constructs of ZNF24, respectively together with viral packaging vectors (pCMV-dR8.9 and pCMV-VSV-G) into HEK293T cells by calcium phosphate. The virus was collected harvested from the supernatant 48 h after transfection, and the target cells were infected with multiplicity of infection (MOI) 20. The cells were screened with puromycin for 48h, and the surviving cells were used for subsequent experiments.

**Patients and tissue samples**

CRC tissues and corresponding non-carcinoma samples (≥5 cm away from the tumor margin) were obtained from a total of 15 patients with colorectal cancer underwent surgical resection at The First Affiliated Hospital of Naval Medical University (Shanghai, China) in March 2022. All the 15 patients were primary colorectal adenocarcinoma, including 2 with mucinous adenocarcinoma. Before surgery, the enrolled patients did not receive any adjuvant therapy. The resected tissue was soaked in liquid nitrogen and stored at −80°C for subsequent analysis. Prior to study enrollment, patients provided written informed consent. The collection of specimens was approved by the Committee on Ethics of Medicine of Naval Medical University (2022-025).

**Plasmid construction**

Plasmid vectors were established according to standard procedures. The ZNF24 overexpression(pcDNA3-ZNF24) and knockdown (pSilencer–siZNF24) vectors were constructed according to standard procedures. The ZNF24 and MMP2 overexpression lentiviral vectors were constructed according to standard procedures. The ZNF24 RNAi lentiviral vector encoding GFP and shRNA targeting ZNF24 was engineered by cloning the following shRNAs into the *Age* I and *EcoR* I restriction site of pMagic7.1 lentiviral plasmid: shZNF24 (sense): 5′- CCGGGGATTTGGAGAGTGAACTTCTCGAGAAGTTCACTCTCCAAATCCTTTTTTG-3′; shZNF24(antisense): 5′- AATTCAAAAAAGGATTTGGAGAGTGAACTTCTCGAGAAGTTCACTCTCCAAATCC-3′; shControl(sense): 5′- CCGGTTCTCCGAACGTGTCACGTTTCAAGAGAACGTGACACGTTCGGAGAATTTTTTG-3′; shControl (antisense): 5′- AATTCAAAAAATTCTCCGAACGTGTCACGTTCTCTTGAAACGTGACACGTTCGGAGAA-3′. The human MMP2 gene promoter (-1964 to +26 bp) was PCR amplified from genomic DNA of HEK293 cells using primers (forward: 5′-CGAGATCTAGGCTGGATTTAGCCCACAGGC-3′; reverse: 5′- AGAAGCTTCCACCGCCTGAGGAAGTCTGGAT -3′) incorporating *Bgl* II and *Hind* III restriction sites, and subcloned into pGL3-Basic. Site-specific mutations were introduced into the putative ZNF24 binding sites (TTTCCATTT) of the MMP2 promoter construct by site-directed mutagenesis. The binding site mutant construct (MMP2‐mut) was generated by PCR mutagenesis using overlapping primers containing the desired mutations (forward: 5′-TGAGATAACTTTTTAAATTTTCACTCTCA -3′; reverse: 5′-TGAGAGTGAAAATTTAAAAAGTTATCTCA -3′). The MMP2 promoter (-1788 to +26 bp) was generated by PCR using primers (forward: 5′- CGAGATCTAAACTGACTCTGGAAAGTCAGAG-3′; reverse: 5′- AGAAGCTTCCACCGCCTGAGGAAGTCTGGAT -3′) and subcloned into pGL3-Basic. All constructs were validated by restriction enzyme analysis and sequencing.

**RNA isolation, cDNA preparation, and quantitative real-time PCR**

Total RNA was extracted from tissues and cells using RNAiso plus reagent (cat.no.9109; Takara). Briefly, PrimeScript RT Reagent kit (cat.no. RR047Q; Takara) was used for cDNA synthesis according to the manufacturer's protocol. To determine the mRNA expression levels of ZNF24 and MMP2, qPCR was performed using TB Green *Premix Ex Taq* II (cat.no. RR820L; Takara) on the StepOne Real‑Time PCR system (Applied Biosystems). The qPCR thermal cycling conditions were as follows: 95˚C for 30 sec, followed by 40 cycles at 95˚C for 10 sec and 60˚C for 40 sec, followed by a final extension step at 72˚C for 2 min. Relative expression values were normalized to GAPDH expression and calculated using the 2^‑ΔΔCt^ method ^51^. The primers used are as follows: ZNF24, forward: 5'- CATTCCCTAAGGCACTGTGAT-3', reverse: 5'-TTGAGGAACACCCATACTGAGA-3'; MMP2, forward: 5'-CCCCAAAACGGACAAAGAG-3', reverse: 5'-CTTCAGCACAAACAGGTTGC-3'; GAPDH, forward: 5'-TGACTTCAACAGCGACACCCA-3', reverse: 5'- CACCCTGTTGCTGTAGCCAAA-3'.

**Western blot**

After preparation in cell lysis buffer containing protease inhibitors, protein lysates were separated by 10% sodium dodecyl sulfate polyacrylamide gel (SDS-PAGE) and incubated with the primary antibody at a diluted concentration according to the manufacture’s protocol. GAPDH was used as a control. The quantifications of bands were conducted by image J. The primary antibodies used were as follows: ZNF24 (cat. no. D224009; Sangon Biotech), MMP2 (cat. no. D198344; Sangon Biotech) and GAPDH (cat. no. D190090; Sangon Biotech).

**Cell proliferation assay**

Cell proliferation tests were performed on days 0, 1, 2, 3, and 4 after transfection according to the CCK-8 manufacturer’s instructions (cat.no. E606335; Sangon Biotech).2000 cells per well were inoculated in 96-well plates and cultured overnight. The CCK-8 reagent was added at the time point and the detection was performed using Synergy 4 Microplate Reader (BioTek).

**Cell cycle assay**

For the cell cycle assay, the Cell Cycle Staining Kit (cat.no. CCS012; Multi Sciences) was used according to the manufacturer's protocol. Briefly, the cells were collected and labelled in the dark at room temperature with propidium iodide stain for 30 minutes. The cells were then measured with FACS Calibur flow cytometer (BD Biosciences) and analyzed with FlowJo software.

**Aging-related β-galactosidase staining assay**

The aging β-Galactosidase Staining Kit (cat.no.C0602; Beyotime Biotechnology) was used for β-galactosidase staining assay according to the instructions.

**Wound healing assay**

The cells were planted into the 24-well plate when cell fusion rate reached 50% to 60%. Gently scrape off the single layer with the tip of 10ul pipette. After scraping, gently rinse the well with the medium to remove the shed cells. The cells were cultured for 72 hours and washed twice by phosphate buffered saline (PBS) and photographed under the microscope.

**Migration and invasion assay**

Cell invasion test was performed using Transwell chambers (cat.no.3422; Corning). Matrigel (cat. no. YZ‑354234; Becton Dickinson) was diluted with serum-free medium, added to the bottom of Transwell chamber and incubated at 37℃ for 1 hour. Briefly, 5×10^4^ cells cultured in serum-free DMEM were seeded onto matrigel‐coated inserts. DMEM with 20% FBS was added into the lower chamber as a chemoattractant and incubated at 37°C. After 24 hours, the non‐invasive cells on the upper surface of the insert were removed by scrubbing. The invasive cells on the bottom surface of the insert were fixed with methanol and stained with crystal violet. Invasiveness was determined by counting the number of invaded cells under a microscope. In the migration assay, matrigel glue was not required, and other experimental steps were the same as in the invasion assay. Each experiment was conducted independently for 2 times.

**Colony formation assay**

Individual cells were suspended into medium. Each group was inoculated with l0 ml medium with 500 cells per dish, and gently shook to make the cells dispersed evenly. Conventional culture for 2 weeks. After the clones were found in the petri dish, the culture medium was cleared, fixed with 4% paraformaldehyde for 15 minutes, stained with 0.1% crystal violet for 10 minutes, washed with PBS, and the number of clones was counted.

**Bioinformatics analysis**

UALCAN (https://ualcan.path.uab.edu/analysis.html) is an online interactive resource, it also provides a convenient access to publicly available cancer omics data, including The Cancer Genome Atlas (TCGA). UALCAN was used to detect the expression level of ZNF24 mRNA in primary colon adenocarcinoma cases using data obtained from TCGA.

**Xenograft assay**

BALB/c nude female mice (age, 4 weeks; weight ~20 g) were purchased from Shanghai SLAC Laboratory Animal Co., Ltd. The animals were housed in an environment with 60% humidity and a temperature of 24˚C, with free access to purified drinking water and food during a 12-hour light and dark cycle. The Committee on Ethics of Medicine of Naval Medical University approved (2022‑025) this study. For tumorigenic models, 2×10^6^ HCT116 cells containing ZNF24 overexpression or vector control and knockdown （siZNF24） or control (siNT) were implanted into the flank of nude mice. Tumor size was measured every 2 days for 8 weeks, and the tumor was measured in three dimensions with a caliper to quantify tumor growth. Tumor volumes were calculated as follows: V (mm^3^) = width^2^ (mm^2^) × length (mm)/2. At the end of the experiment, all animals were euthanized, tumors were excised and weighed. For experimental liver metastasis, 1×10^6^ HCT116 cells containing ZNF24 overexpression or vector control and knockdown (siZNF24) or control (siNT) were injected into the spleen of mice. After 6 weeks, the liver was examined for liver metastasis and tumors were counted.

**MMP2 promoter reporter assay**

HEK293T cells were cultured in antibiotic-free medium for 24 hours, then cotransfected with equimolar ratios of either a pcDNA3-ZNF24 or pcDNA3 expression vector (pSilencer-siZNF24 or pSilencer 1.0-U6 siRNA expression Vector) and with either MMP2 promoter luciferase constructs or a control vectors using Xfect transfection reagent (cat.no. 631317; Clontech) according to the manufacturer’s protocol. After 48 hours, the cells were lysed with Passive lysis buffer (cat.no. E1910; Promega) according to the manufacturer’s protocol. The luciferase activity in lysate was evaluated by the Dual luciferase reporter assay system (cat.no. E1910; Promega) according to the manufacturer’s protocol. The results were normalized to the transfection efficiency of a third co-transfected thymidine kinase driven renilla luciferase vector.

**Statistical analysis**

Data are presented as the mean ± standard deviation (SD). Student's *t*-tests and Chi-square test were used to compare differences when appropriate. Statistical analysis was performed using Prism 9 (GraphPad).

**
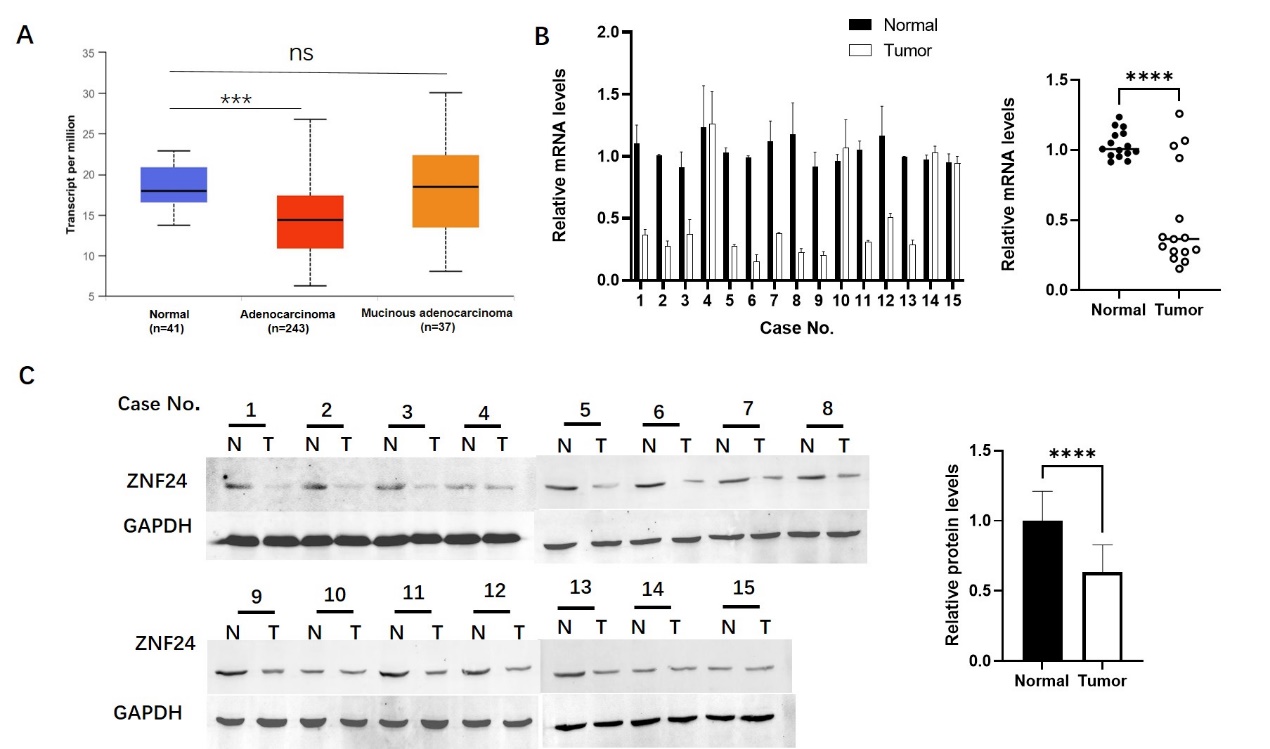
**

**Fig S1** ZNF24 is down-regulated in colorectal cancer. (A) The expression levels of ZNF24 in colorectal adenocarcinoma (243 cases), mucinous adenocarcinoma (37 cases) and normal tissues (41 cases) were analyzed by UALCAN. (B) ZNF24 mRNA levels in colorectal cancer and adjacent normal tissues were analyzed by RT-qPCR in 15 cases (13 cases of colorectal adenocarcinoma and 2 cases of mucinous adenocarcinoma with 4 and 10). (C) Western blot analysis of ZNF24 protein levels in 15 cases of colorectal cancer (T) and adjacent normal (N) tissues. ***P<.001, ****P<.0001.


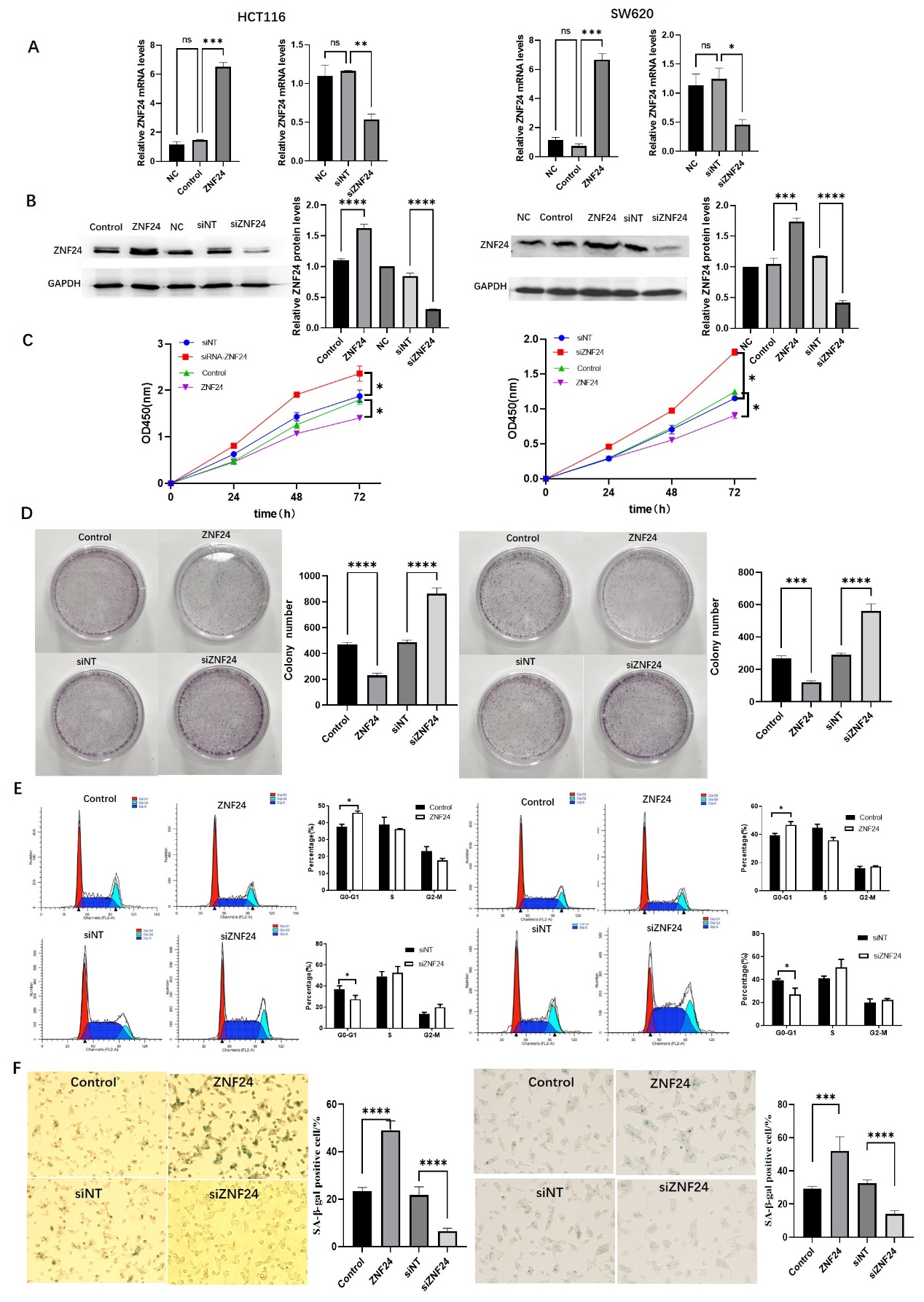


**Fig S2** ZNF24 inhibited colorectal cancer cell growth *in vitro*. HCT116 and SW620 cells were infected with ZNF24 overexpression and interference lentivirus for 48 hours, respectively. The expression level of ZNF24 was analyzed by RT-qPCR (A) Western blot (B). The effect of ZNF24 overexpression and knockdown on HCT116 or SW620 cell growth was measured with CCK-8 assay (C) and with colony formation assay (D). (E) Flow cytometric analysis was used to analyze cell cycle distribution and statistics of cell cycle distribution. ZNF24 induces G0/G1 cell cycle arrest in HCT116 and SW620 cells. （F）ZNF24 induces senescence of HCT116 and SW620 cells. The activity of aging-related β-galactosidase (left) and the related statistics (right) were analyzed to determine the aging cells. *P<.05, ***P<.001, ****P<.0001.

**
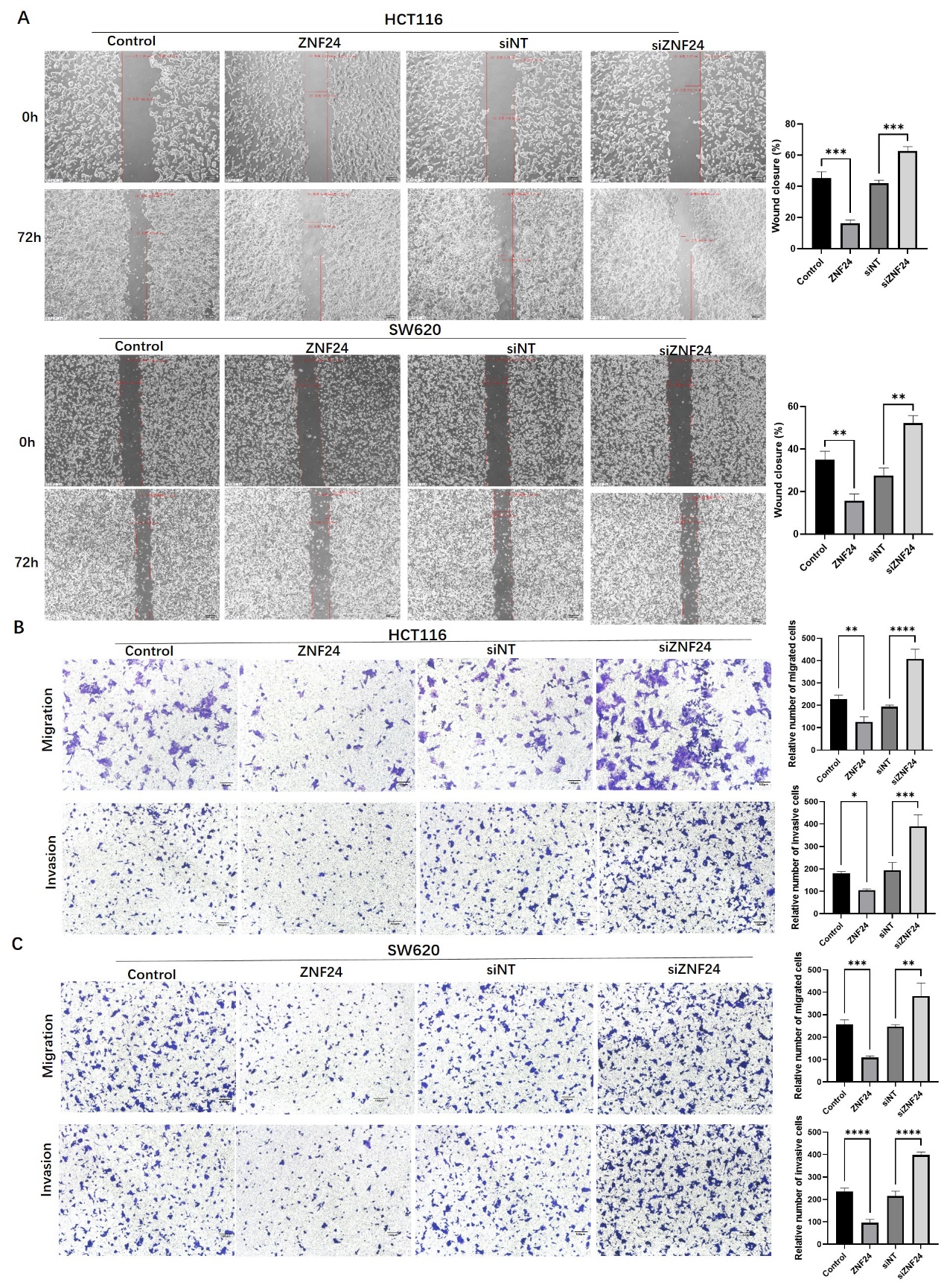
**

**Fig S3** ZNF24 inhibits the migration and invasion of HCT116 and SW620 cells. (A) Representative wound healing images of HCT116 and SW620 cells either overexpressing ZNF24 or knocking down ZNF24; scale bar, 200 μm. (B) Representative transwell migration /invasion images and quantification numbers of HCT116 cells either overexpressing ZNF24 or knocking down ZNF24; scale bar, 100 μm. (C) Representative transwell migration /invasion images and quantification numbers of SW620 cells either overexpressing ZNF24 or knocking down ZNF24; scale bar, 100 μm; **P < .01, ***P < .001.

**
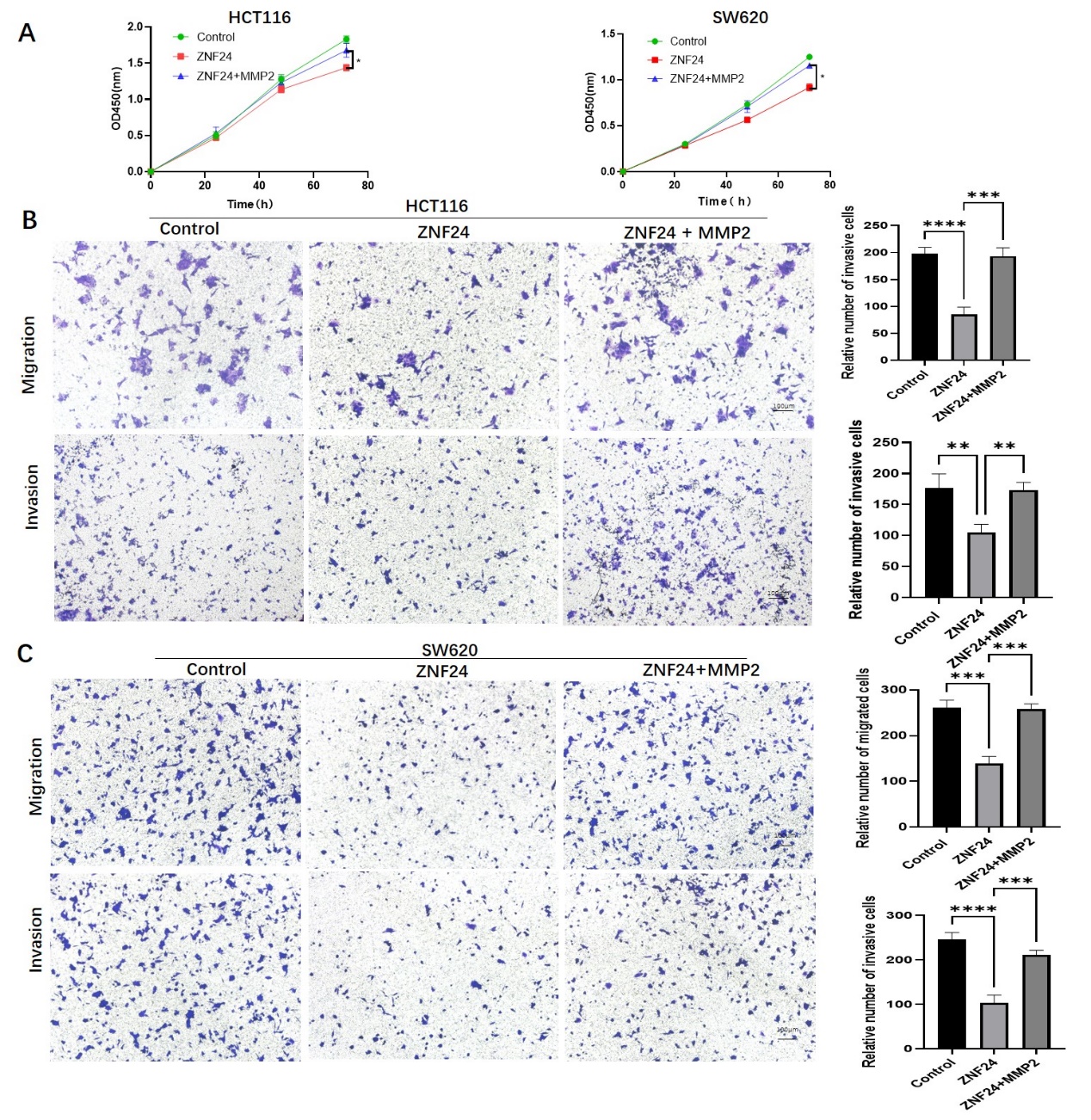
**

**Fig S4** MMP2 overexpression rescue ZNF24-mediated CRC cell proliferation, migration and invasion. (A) The effect of MMP2 overexpression on ZNF24-overexpressing HCT116 or SW620 cell growth was measured with CCK-8 assay. (B) Representative transwell migration and invasion images and quantification numbers of MMP2 overexpression in ZNF24-overexpressing HCT116 cells; scale bar, 100 μm. (C) Representative transwell migration and invasion images and quantification numbers of MMP2 overexpression in ZNF24-overexpressing SW620 cells; scale bar, 100 μm. *P<.05, **P<.01, ***P<.001, ****P<.0001.
